# Diagnostic value of thyroid transcription factor-1 for pleural or other serous metastases of pulmonary adenocarcinoma: a meta-analysis

**DOI:** 10.1038/srep19785

**Published:** 2016-01-25

**Authors:** Yongchun Shen, Caishuang Pang, Konglong Shen, Yanqiu Wu, Diandian Li, Chun Wan, Zenglin Liao, Ting Yang, Lei Chen, Fuqiang Wen

**Affiliations:** 1 Department of Respiratory and Critical Care Medicine, West China Hospital of Sichuan University and Division of Pulmonary Diseases, State Key Laboratory of Biotherapy of China, Chengdu 610041, China; 2 Radiation Physics Center, Cancer Center and State Key Laboratory of Biotherapy, West China Hospital of Sichuan University, Chengdu 610041, China

## Abstract

The role of thyroid transcription factor 1 (TTF-1) in the diagnosis of metastatic pulmonary adenocarcinomas in pleural, pericardial, and peritoneal effusions has not been defined. This study aimed to assess the overall diagnostic accuracy of TTF-1 for metastatic pulmonary adenocarcinomas in pleural or other effusions. Literature search was conducted in PubMed, EMBASE, and other databases to find eligible publications. Quality was assessed according to standardized QUADAS-2 criteria. Sensitivity, specificity, positive/negative likelihood ratio (PLR/NLR), and diagnostic odds ratio (DOR) were pooled. Summary receiver operating characteristic (SROC) curves were used to assess overall performance of the TTF-1 assay. A systematic search revealed 20 studies comprising a total of 1,213 subjects in this meta-analysis. The summary estimates were listed as follows: sensitivity, 0.74 (95% CI: 0.69–0.79); specificity, 0.99 (95% CI: 0.97–1.00); PLR, 78.16 (95% CI: 27.15–225.05); NLR, 0.26 (95% CI: 0.22–0.32); and diagnostic odds ratio, 297.75 (95% CI: 104.16–851.19). Estimated positive and negative post-probability values for metastatic pulmonary adenocarcinomas prevalence of 20% were 95% and 6%, respectively. The area under the SROC curve was 0.96. TTF-1 shows significant potential as a diagnostic marker to differentiate metastatic pulmonary from non-pulmonary adenocarcinomas in pleural or other effusions. These results justify larger, more rigorous studies to confirm such a diagnostic role.

Adenocarcinoma has become the most common histological type of lung cancer, approximately half of lung cancer patients are diagnosed with adenocarcinoma, and the incidence is rapidly increasing worldwide^1^,^2^. Metastatic pulmonary adenocarcinomas often manifest as pleural effusion, while some cases present as pericardial, or peritoneal effusions^3^. It is important to identify the primary site of adenocarcinomas in pleural, pericardial, and peritoneal effusions, since this information guides disease treatment and management, as well as prognosis assessment^4^. However, identifying adenocarcinoma origin can be challenging; for example, it is difficult or even impossible to differentiate metastatic pulmonary adenocarcinomas from non-pulmonary adenocarcinomas on the basis of morphology and effusion samples^5^. Positive cytology examination may suggest the presence of malignant diseases, but it may not indicate the primary site of adenocarcinoma.

Immunostaining can help identify the site of origin, but most adenocarcinoma markers are not organ-specific. This highlights the need for more biologically specific markers for pulmonary adenocarcinomas in order to distinguish metastatic pulmonary from non-pulmonary adenocarcinomas in pleural, pericardial, and peritoneal effusions^6^,^7^. Thyroid transcription factor 1 (TTF-1) is a homeodomain-containing transcription factor selectively expressed in the thyroid, diencephalon and lung^8^. TTF-1 was recently proposed as an immunohistochemical marker of pulmonary adenocarcinomas, with one meta-analysis reporting overall sensitivity of 76% and specificity of 100% in tissue samples^9^. TTF-1 also plays a role in the diagnosis of malignant effusion^10^. Studies suggest that TTF-1 is a potential biomarker for differentiating pulmonary adenocarcinomas from non-pulmonary adenocarcinomas in pleural or other effusions^11^,^12^,^13^, but results from these studies have not always been consistent. Therefore we meta-analyzed the available evidence on whether TTF-1 can distinguish metastatic pulmonary adenocarcinomas from non-pulmonary adenocarcinomas in pleural or other effusions.

## Material and Methods

This meta-analysis was conducted and reported according to the guidelines of the Preferred Reporting Items for Systematic Reviews, the Meta-analysis Statement and methods recommended by the Cochrane Diagnostic Test Accuracy Working Group^14^,^15^. There was no need for institutional review board approval for this retrospective meta-analysis.

### Literature search

Two investigators (Y. Shen and C. Pang) searched in PubMed, EMBASE, Web of Knowledge, CNKI, WANFANG, and WEIPU databases for relevant articles published up to May 2015. The following search terms were used as Medical Headings and/or text words: “Thyroid transcription factor 1 OR TTF-1” AND “pleural effusion OR pleural fluid OR hydrothorax OR ascites OR peritoneal effusion OR pericardial effusion OR serous effusion” AND “sensitivity OR specificity OR accuracy”. Reference lists of the included studies and review articles were also checked to identify additional studies.

### Selection of eligible studies

A study was included if it fulfilled the following criteria: (i) it examined the ability of TTF-1 to differentiate metastatic pulmonary adenocarcinomas from non-pulmonary adenocarcinomas in humans; (ii) it analyzed pleural, pericardial, and peritoneal effusions as samples; (iii) it reported sufficient data to allow calculation of true positive (TP), false positive (FP), false negative (FN), and true negative (TN) rates; (iv) it reported definitive determination of metastatic pulmonary adenocarcinomas and non-pulmonary adenocarcinomas using gold-standard methods; and (v) it was an original research study published in English or Chinese. Conference proceedings and studies published only as abstracts were excluded. To avoid selection bias, we also excluded studies involving fewer than 20 patients. When several articles concerned the same subjects, only results from the publication with the largest sample were used.

### Data extraction

Two reviewers (Y. Shen and C. Pang) independently identified eligible studies and extracted data on study methodology, characteristics and test accuracy using a standardized extraction form. The data extracted were: name of first author, publication year, country, serous effusion types, sample preparation method, TTF-1 immunostaining method, antibody clone and dilution, and two-by-two tables of TP, TN, FP and FN. Detailed information about controls with non-pulmonary adenocarcinoma was also reviewed.

### Assessment of methodological quality

The same two reviewers (Y. Shen and C. Pang) assessed the quality of the selected studies using the Quality Assessment of Diagnostic Accuracy Studies-2 (QUADAS-2) criteria, which cover four key domains for assessing risk of bias and applicability of the study results. These domains are patients selection, index test, reference standard, and flow and timing of samples/patients through the study^16^. Any discrepancies between the two authors (Y. Shen and C. Pang) during study selection, data extraction or quality assessment were resolved by discussion with a third author (K. Shen).

### Statistical analysis

We used standard methods recommended for bivariate meta-analysis of diagnostic test evaluations^17^. We descriptively analyzed study characteristics and QUADAS-2 quality assessment using Excel and Review Manager 5.2 (The Cochrane Collaboration, Copenhagen, Denmark). The following measures of test accuracy were computed for each study, together with 95% confidence intervals (95% CIs): sensitivity, specificity, positive likelihood ratio (PLR), negative likelihood ratio (NLR) and diagnostic odds ratio (DOR). A summary ROC (SROC) curve covering all the studies was plotted using the data on sensitivity and specificity for a single test threshold from each study. The area under the SROC curve (AUC) was used to summarize the overall diagnostic performance of TTF-1.

The heterogeneity effect was measured using the Q test and the inconsistency index (I^2^). P < 0.05 or I^2^ ≥ 50% indicated significant heterogeneity, which was then analyzed through meta-regression to identify potential covariates. Deeks’s funnel plot was used to detect publication bias^18^. Post-test probability was calculated using the overall prevalence of 20% with Fagan nomograms. All analyses were performed using the “Midas” module in STATA 12.0 (Stata Corp., College Station, TX), and Meta-DiSc 1.4 for Windows (XI, Cochrane Colloquium, Barcelona, Spain). All statistical tests were two-sided, with P < 0.05 taken as the threshold for statistical significance.

## Results

**Figure 1:**
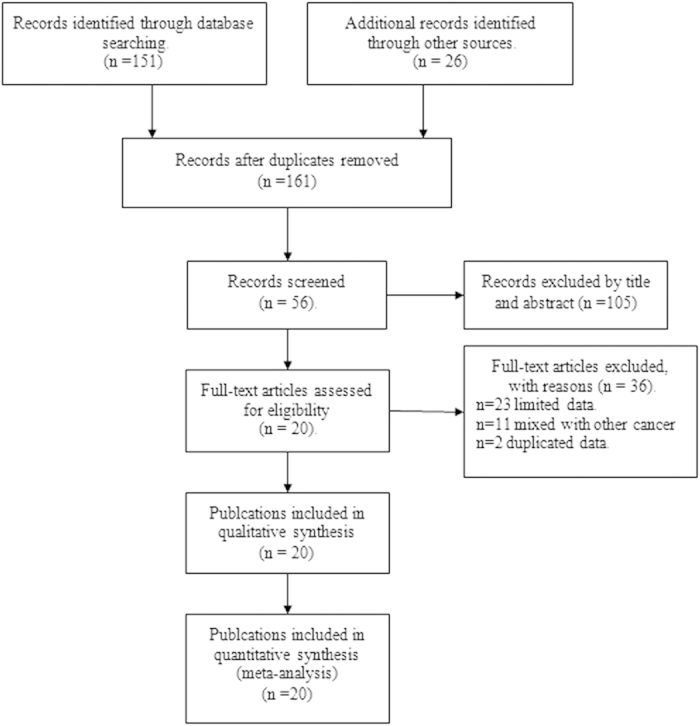
Flow diagram of literature search.

Systematically searching literature databases and manually searching reference lists in relevant reviews and studies identified 20 studies examining the diagnostic accuracy of TTF-1 in pleural or other effusions in patients with metastatic pulmonary adenocarcinomas^19^,^20^,^21^,^22^,^23^,^24^,^25^,^26^,^27^,^28^,^29^,^30^,^31^,^32^,^33^,^34^,^35^,^36^,^37^,^38^. Studies were excluded because they were not diagnostic studies, they did not report sufficient data to construct 2 × 2 tables, or they mixed other type of cancers like squamous-cell carcinoma. The process of selecting of studies eligible for inclusion is shown in Fig. 1.

### Patient characteristics and study design

The final set of 20 studies involved 1,213 subjects, comprising 668 patients with metastatic pulmonary adenocarcinomas and 545 controls with non-pulmonary adenocarcinomas (median 60 patients per study; range 32–113 patients). (Table 1). Thirteen studies were performed in Asia, six in the USA, and one in Europe. The most frequent cancer types among the 545 patients with non-pulmonary adenocarcinomas were breast (n = 178), gastrointestinal (n = 147) and ovary adenocarcinomas (n = 145). Eight studies assayed pleural effusion; four studies, pleural effusion, pericardial effusion, and ascites; another four studies, pleural effusion and ascites; two studies, only mentioned as serous effusions; one study, pleural effusion and pericardial effusion; and one study, only pericardial effusion. Only two studies involved analysis of smear samples^25^,^34^; the remainder relied on analysis of cell blocks. Table 2 summarizes individual study designs and results for diagnostic performance of TTF-1.

Most studies detected TTF-1 using the 8G7G3/1 antibody; three used the SPT24 antibody^33^,^34^,^37^, and one study did not report this information^35^. Twelve studies used immunohistochemistry to detect TTF-1, while the remaining eight used immunocytochemistry. Antibody dilutions from 1:40 to 1:500 were used in the included studies, while five did not report dilution factors. All studies defined nuclear staining as positive. Supplemental Table 1 summarizes the clinical information of patients with non-pulmonary adenocarcinomas.

### Methodological quality of the included studies

**Figure 2:**
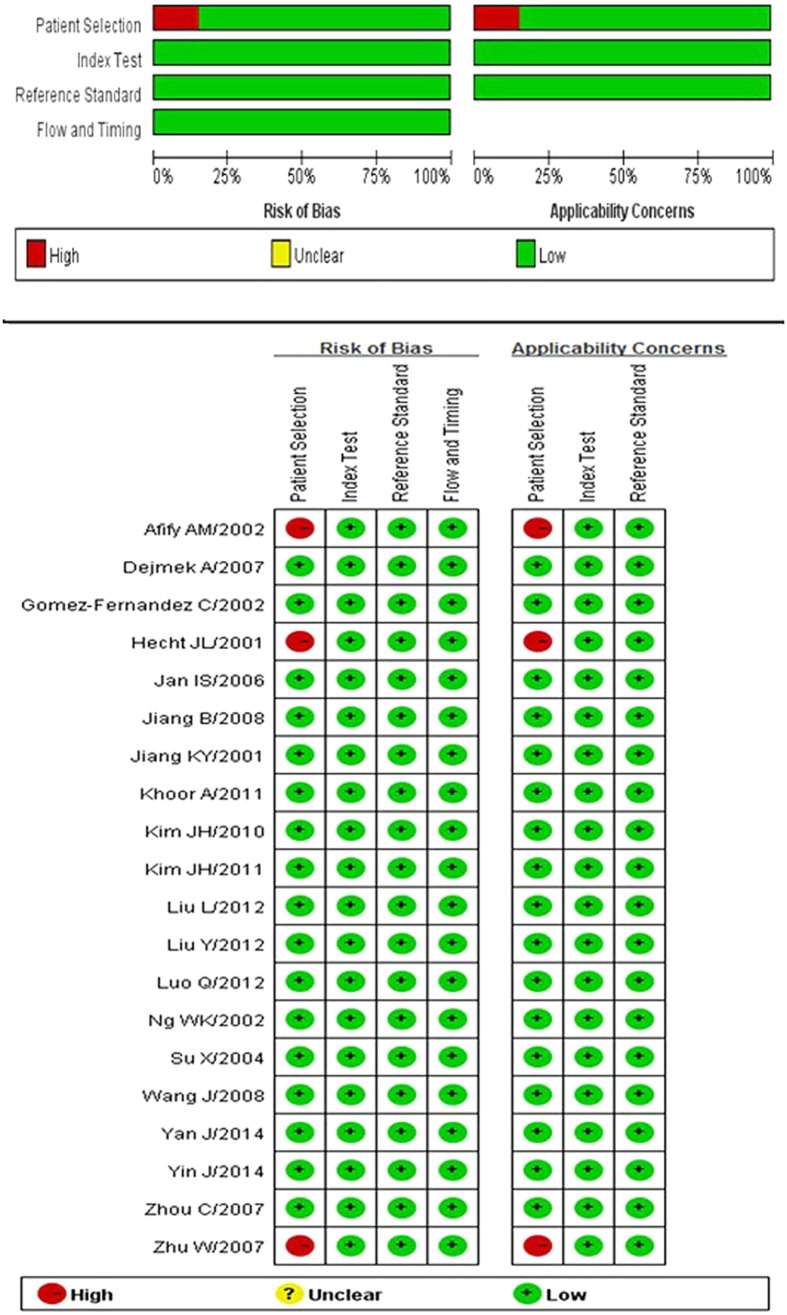
Summary of QUADAS-2 assessments of included studies. QUADAS-2: Quality Assessment of Diagnostic Accuracy Studies-2. Patient Selection: Describe methods of patient selection; Index Text: Describe the index test and how it was conducted and interpreted; Reference Standard: Describe the reference standard and how it was conducted and interpreted; Flow and Timing: Describe any patients who did not receive the index tests or reference standard or who were excluded from the 2 × 2 table, and describe the interval and any interventions between index tests and the reference standard. (From *Ann Intern Med.* 2011; 155(8):529–36.)

QUADAS-2 was proposed in 2011 as an improved redesign of the original QUADAS and it was integrated into RevMan 5.2 in 2012. We applied the four criteria of QUADAS-2 (patient selection, index test, reference standard, flow and timing) to the studies in our meta-analysis. A response of “Yes” was given if the criterion was fulfilled, “Unclear” if fulfillment was unclear, and “No” if the criterion was not fulfilled. Based on these responses, the risk of bias for each criterion was classified as low, high, or unclear. Based on the first three domains, the applicability of the results was also evaluated. The quality of included studies was generally good, but three studies^19^,^21^,^27^ were at high risk of bias due to deficiencies in patient selection. Figure 2 shows the summary of QUADAS-2 assessments of included studies.

### Diagnostic accuracy of TTF-1

**Figure 3:**
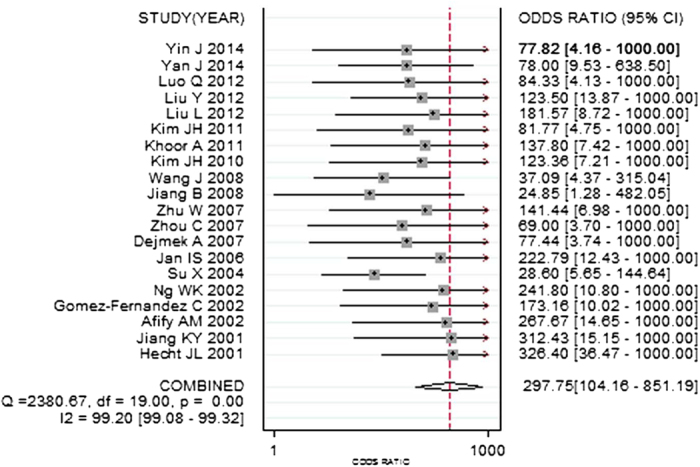
Forest plots of diagnostic odds ratio of thyroid transcription factor-1. The pooled diagnostic odds ratio was 297.75 (95% CI: 104.16–851.19).

Sensitivity of TTF-1 for diagnosing metastatic pulmonary adenocarcinomas in effusions was between 0.54 and 0.88, and the pooled sensitivity was 0.74 (95% CI: 0.69–0.79). Specificities of TTF-1 varied from 0.92 to 1.00, and the pooled specificity was 0.99 (95% CI: 0.97–1.00). The other pooled parameters for TTF-1, calculated over all 20 studies, were: PLR, 78.16 (95% CI: 27.15–225.05); NLR, 0.26 (95% CI: 0.22–0.32); and DOR, 297.75 (95% CI: 104.16–851.19) (Fig. 3).

**Figure 4:**
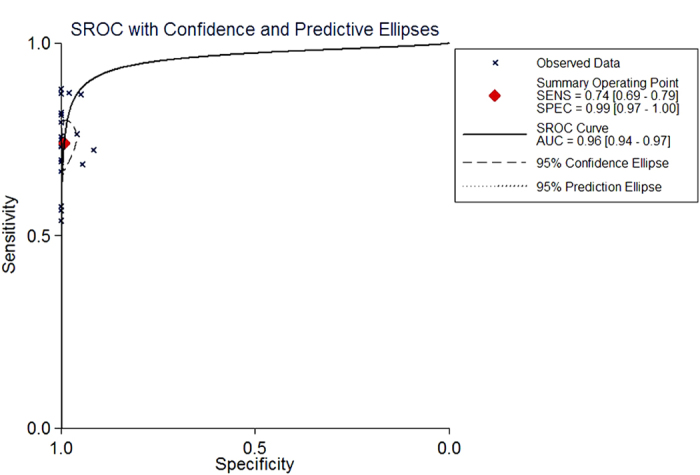
Summary receiver operating characteristic (SROC) curves for the detection of metastatic pulmonary adenocarcinoma using thyroid transcription factor-1. The SROC curve with confidence and prediction regions around mean operating sensitivity and specificity point analyses of TTF-1. AUC, area under the curve; SENS, sensitivity; SPEC, specificity.

**Figure 5:**
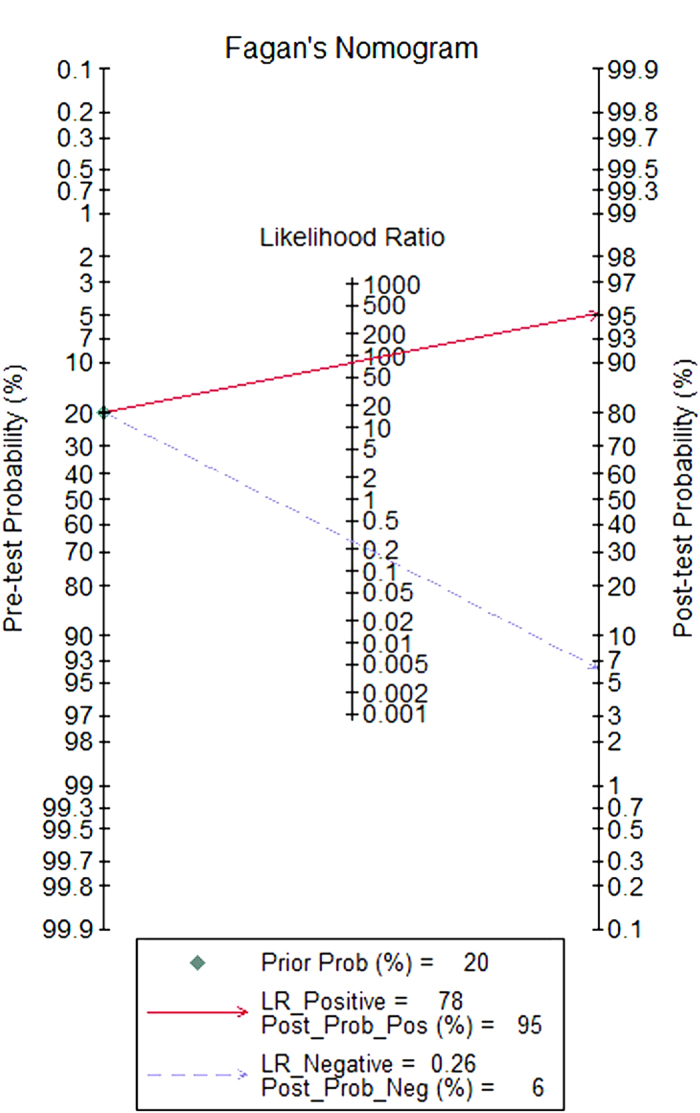
Fagan plot analysis to evaluate the clinical utility of thyroid transcription factor-1 for the detection of metastatic pulmonary adenocarcinoma.

Figure 4 shows a plot of the TP rate as a function of the FP rate in individual studies, as well as the corresponding SROC curve. The AUC was 0.96, indicating a high discriminatory ability for TTF-1. Fagan’s nomogram for likelihood ratios (Fig. 5) indicated that using TTF-1 to detect metastatic pulmonary adenocarcinomas increased the post-probability to 95% when the results were positive, and reduced the post-probability to 6% when the results were negative.

### Meta-regression and publication bias

I^2^ values for diagnostic performance indices were as follows: sensitivity, 51.7% (P = 0.00); specificity 30.2% (P = 0.10); PLR 0.00% (P = 0.16); NLR, 57.57% (P = 0.00); and DOR, 99.20% (P = 0.00). This suggests high heterogeneity among included studies, so, a meta-regression was performed to identify possible sources of heterogeneity. The meta-regression featured six covariates: (i) country of origin (Asia vs. non-Asia); (ii) TTF-1 assay method (immunohistochemistry vs. immunocytochemistry), (iii) TTF-1 clone (8G7G3/1 vs. other); (iv) TTF-1 antibody dilution (≤1:150 vs. >1:150 and other), (v) study design (prospective vs. retrospective); and (vi) blinding (blind vs. other). None of these covariates was found to be a significant source of heterogeneity (all P > 0.05, Table 3).

**Figure 6:**
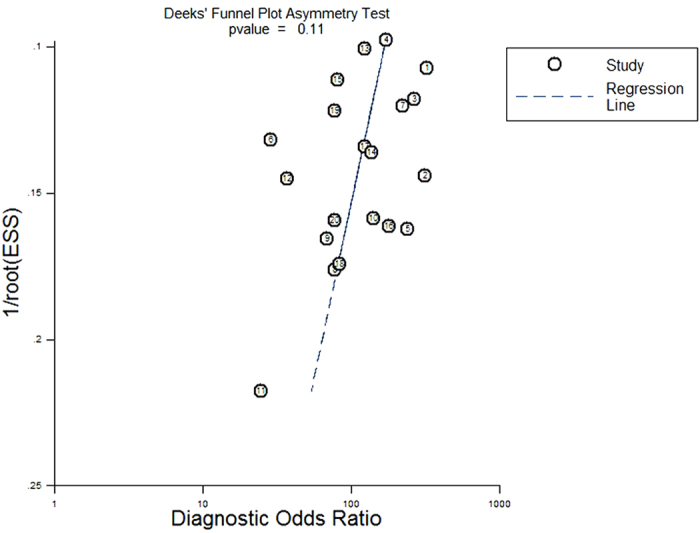
Deeks’s funnel plot to assess the likelihood of publication bias. The statistically non-significant P-value of 0.11 for the slope coefficient suggests symmetry in the data and a low likelihood of publication bias.

Deeks’s funnel plot asymmetry test was used to assess likelihood of publication bias in the included 20 studies. The slope coefficient was associated with P = 0.11, suggesting symmetry in the data and low likelihood of such bias (Fig. 6).

## Discussion

Diagnosing lung adenocarcinoma based on resection histology is normally straightforward, but diagnosing metastatic pulmonary adenocarcinomas based on effusion samples can be extremely challenging^5^,^6^. Available biomarkers in effusions differentiate poorly between pulmonary adenocarcinomas and non-pulmonary adenocarcinomas. TTF-1 has emerged as a promising candidate biomarker, but studies of its diagnostic performance have given conflicting results^11^,^12^,^13^. Our meta-analysis is strengthened by the use of a standard protocol, strict inclusion criteria, standardized data extraction, independent reviewers, and a bivariate random-effects model^39^. Our meta-analysis of available evidence suggests that TTF-1 can accurately predict whether an adenocarcinoma cell originated from a pulmonary or non-pulmonary site. However, TTF-1 probably cannot stand on its own and so should be used in conjunction with other markers.

Our meta-analysis indicated that TTF-1 performed with medium sensitivity (0.74, 95% CI: 0.69–0.79); and high specificity (0.99, 95% CI: 0.98–1.00), with a relatively high rate of missed diagnoses (16%) but a low rate of misdiagnosis (1%). These findings suggest that TTF-1 is a highly specific marker of pulmonary adenocarcinoma origin in pleural and other effusions. The SROC curve, which assesses overall test performance by showing the trade-off between sensitivity and specificity^40^, had an AUC of 0.96, suggesting high overall accuracy. Another indicator of diagnostic accuracy is DOR, which combines sensitivity and specificity data into a single number ranging from 0 to infinity, with higher values indicating better discriminatory test performance. Mean DOR in our meta-analysis was 297.75, suggesting that assaying TTF-1 should be helpful in the diagnosis of metastatic pulmonary adenocarcinomas. We further examined the diagnostic accuracy of TTF-1 by calculating PLR and NLR, which can be easier to relate to clinical practice than SROC and DOR. The pooled PLR value of 78.16 suggests that patients with metastatic pulmonary adenocarcinomas have an approximately 78-fold higher chance of giving a positive TTF-1 result than do patients without metastatic pulmonary adenocarcinomas. At the same time, the pooled NLR was 0.26, indicating that a negative TTF-1 result is still 26% likely to be a false negative, which is not low enough to rule out metastatic pulmonary adenocarcinomas.

The relatively low sensitivity of TTF-1 in identifying metastatic pulmonary adenocarcinoma cells in effusion samples means that it is probably not sufficiently reliable on its own. Instead it should be used in conjunction with other markers. For example, combining of TTF-1 and napsin A gave higher sensitivity and accuracy than TTF-1 alone in identifying metastatic pulmonary adenocarcinomas^34^. Carcino-embryonic antigen is often targeted during immunostaining of metastatic pulmonary adenocarcinoma in pleural or other effusions^41^, so, including TTF-1 within a panel of immunostaining markers such as CEA, and napsin A, may increase the overall sensitivity and specificity, thereby improving overall accuracy.

Though TTF-1 may play a role in identifying malignant effusions, comparing the diagnostic performance of TTF-1 with that of classical tumor markers such as CA15-3 and vascular endothelial growth factor (VEGF) is difficult, because the two types of biomarker serve different purposes. Immunostaining for TTF-1 is done primarily to determine the source of malignant cells. When measured by ELISA on pleural fluid supernatants, TTF-1 had a poor diagnostic accuracy for differentiating malignant and benign effusions with the sensitivity of only 9%^10^. Examining markers such as CA 15-3 and VEGF, or using other diagnostic tools such as percutaneous pleural biopsy and VATS-directed biopsy, is done to determine whether effusions are malignant or benign^42^,^43^,^44^,^45^.

TTF-1 is also a sensitive marker for papillary carcinoma of the thyroid, although it is estimated that fewer than 1% of patients with papillary thyroid carcinoma have malignant pleural effusions^46^. In this meta-analysis, only one case of thyroid carcinoma was reported in 545 patients with non-pulmonary adenocarcinomas. The rarity of metastatic thyroid carcinoma in serous effusions explains the nearly 100% specificity of TTF-1 in detecting metastatic lung adenocarcinoma across several studies.

Our meta-analysis results indicated an association between TTF-1 and presence of metastatic pulmonary adenocarcinomas, implying that TTF-1 may contribute to such metastasis. Winslow *et al.* reported that downregulation of TTF-1 is associated with loss of differentiation, enhanced tumor seeding ability and increased metastatic potential in lung adenocarcinoma^47^. Positive and partially positive TTF-1 expression in lung adenocarcinoma patients correlates with EGFR mutations (exon 19 and 21). In clinical practice, the combination of TTF-1 expression and EGFR mutations, especially mutations in exon 21, can guide timely clinical treatment for lung adenocarcinomas^48^. Future studies should examine how TTF-1 functions in lung adenocarcinoma-related regulatory and signaling pathways. At the same time, researchers and clinicians should not overextend their interpretations of TTF-1 expression, which should be taken into account only when malignant cells are present. Indeed, the requirement for malignant cells limits the diagnostic sensitivity and clinical significance of TTF-1, and distinguishes it from assays based on circulating tumor DNA or classical tumor markers.

Standardized techniques for detecting TTF-1 should be established in order to maximize the clinical utility of this biomarker. Studies should rigorously determine whether immunohistochemistry or immunocytochemistry is superior, and the dilution of primary antibody should be optimized. Dilution factors among the studies in this meta-analysis ranged from 1:40 to 1:500. Studies should also compare the different primary antibodies available; one study has suggested that the SPT24 antibody clone is better than the 8G7G3/1 clone^49^. It may also be possible to improve sensitivity or specificity of immunohistochemical staining by optimizing antibody cut-off values^50^.

The findings of this meta-analysis should be interpreted with caution because of several limitations. While our strict inclusion and exclusion criteria may have helped reduce selection bias, they led to a relatively small final set of studies for which statistical power may be inadequate for drawing definitive conclusions about the ability of TTF-1 to discriminate metastatic pulmonary adenocarcinomas from metastatic non-pulmonary adenocarcinomas in pleural or other effusions. For example, we included only studies published in English and Chinese in a relatively small number of databases. Our results may be biased by our omission of unpublished studies, studies published in other languages and studies published in journals not indexed in the databases we searched. In addition, we detected substantial heterogeneity across the included studies, for which we were unable to identify causes using meta-regression. Future studies should aim for greater rigor in order to decrease the risk of bias.

## Conclusions

In summary, our meta-analysis suggests that TTF-1 may significantly aid the diagnosis of metastatic pulmonary adenocarcinomas in pleural or other effusions. Our data provide further evidence that TTF-1 is a useful marker for distinguishing metastatic adenocarcinoma of the lung from non-pulmonary adenocarcinoma in specimens of pleural or other effusions.

## Additional Information

**How to cite this article**: Shen, Y. *et al.* Diagnostic value of thyroid transcription factor-1 for pleural or other serous metastases of pulmonary adenocarcinoma: a meta-analysis. *Sci. Rep.* **6**, 19785; doi: 10.1038/srep19785 (2016).

## Supplementary Material

Supplemental Table 1

## Figures and Tables

**Table 1:** Clinical summary of included studies.

Author (Ref)	Year	Country	Subjects	Samples	Sample	TTF-1	TTF-1	Dilution	Cut-off
					preparation	assay	Clone		
Hecht JL^15^	2001	USA	88	PE,PTE,PAE	Cell block	IHC	8G7G3/1	1:200	Nuclear staining
Jiang KY^16^	2001	Korea	56	PE,PTE	Cell block	IHC	8G7G3/1	NA	Nuclear staining (≥10% cells)
Afify AM^17^	2002	USA	70	SF	Cell block	IHC	8G7G3/1	1:40	Nuclear staining
Gomez-Fernandez C^18^	2002	USA	113	PE,PTE	Cell block	ICC	8G7G3/1	1:150	Nuclear staining (≥10% cells)
Ng WK^19^	2002	China	36	PE,PTE,PAE	Cell block	IHC	8G7G3/1	1:50	Nuclear staining
Su X^20^	2004	China	60	PE,PTE,PAE	Cell block	ICC	8G7G3/1	RTU	Nuclear staining
Jan IS^21^	2006	China	75	PE,PAE	Smear	ICC	8G7G3/1	1:200	Nuclear staining (≥10% cells)
Dejmek A^22^	2007	Sweden	32	PE	Cell block	IHC	8G7G3/1	1:100	Nuclear staining
Zhou C^23^	2007	China	48	PE	Cell block	IHC	8G7G3/1	1:100	Nuclear staining
Zhu W^24^	2007	USA	46	SF	Cell block	IHC	8G7G3/1	1:200	Nuclear staining
Jiang B^25^	2008	China	48	PE	Cell block	ICC	8G7G3/1	NA	Nuclear staining (≥10% cells)
Wang J^26^	2008	China	53	PE	Cell block	IHC	8G7G3/1	NA	Nuclear staining
Kim JH^27^	2010	Korea	97	PE,PTE	Cell block	ICC	8G7G3/1	1:50	Nuclear staining
Khoor A^28^	2011	USA	52	PE	Cell block	ICC	8G7G3/1	1:500	Nuclear staining
Kim JH^29^	2011	Korea	84	PE	Cell block	IHC	8G7G3/1	1:50	Nuclear staining
Liu L^30^	2012	USA	38	PE	Smear/Cell block	IHC	SPT24	RTU	Nuclear staining (≥10% cells)
Liu Y^31^	2012	China	65	PE,PTE,PAE	Cell block	IHC	SPT24	NA	Nuclear staining (≥5% cells)
Luo Q^32^	2012	China	31	PE,PTE	Cell block	ICC	NA	NA	Nuclear staining (≥10% cells)
Yan J^33^	2014	China	76	PE	Cell block	ICC	8G7G3/1	1:50	Nuclear staining (≥5% cells)
Yin J^34^	2014	China	45	PAE	Cell block	IHC	SPT24	RTU	Nuclear staining

ICC: Immunocytochemistry; IHC: Immunohistochemistry; NA: Not available; PAE: pericardial effusion; PE: Pleural effusion; PTE: peritoneal effusion; RTU: Ready to use; TTF-1: Thyroid transcription factor 1.

**Table 2:** Diagnostic performance of thyroid transcription factor-1 and design from individual studies.

Author (Ref)	Cases	Controls	TP	FP	FN	TN	Design	Blinded?
Hecht JL^15^	39	49	34	1	5	48	NA	NA
Jiang KY^16^	16	40	13	0	3	40	R	NA
Afify AM^17^	34	36	27	0	7	36	R	NA
Gomez-Fernandez C^18^	39	74	21	0	18	74	R	Yes
Ng WK^19^	17	19	15	0	2	19	R	NA
Su X^20^	36	24	26	2	10	22	R	NA
Jan IS^21^	50	25	41	0	9	25	R	Yes
Dejmek A^22^	12	20	8	0	4	20	P	Yes
Zhou C^23^	37	11	28	0	9	11	NA	NA
Zhu W^24^	13	33	9	0	4	33	R	Yes
Jiang B^25^	43	5	30	0	13	5	P	Yes
Wang J^26^	35	18	24	1	11	17	R	NA
Kim JH^27^	52	45	30	0	22	45	P	Yes
Khoor A^28^	26	26	19	0	7	26	R	Yes
Kim JH^29^	53	31	30	0	23	31	R	Yes
Liu L^30^	23	15	20	0	3	15	R	Yes
Liu Y^31^	45	20	39	1	6	19	R	Yes
Luo Q^32^	15	16	11	0	4	16	R	NA
Yan J^33^	51	25	39	1	12	24	R	NA
Yin J^34^	32	13	24	0	8	13	R	NA

NA: Not available; P: Prospective; R: retrospective; FN: False negative; FP: False positive; TN: True negative; TP: True positive;

**Table 3:** Mata-regression of potential heterogeneity within the included studies.

Covariates	Number of studies	Coefficient	SE	RDOR(95%CI)	P value
Country					
Asian	13	−0.654	0.7549	0.52(0.10–2.69)	0.4032
non-Asian	7				
TTF-1 assay method					
IHC	12	0.372	0.6542	1.45(0.35–6.04)	0.58
ICC	8				
TTF-1 clone					
8G7G3/1	16	−0.296	0.9242	0.74(0.10–5.57)	0.7543
Other	4				
TTF-1 dilution					
≤1:150	8	0.216	0.7699	1.24(0.23–6.64)	0.7843
1:150 and other	12				
Study Design					
Prospective	4	−0.617	1.0947	0.54(0.05–5.86)	0.5835
Retrospective and NA	16				
Blinding					
Yes	10	0.1	0.7958	1.11(0.20–6.26)	0.9016
No and NA	10				

ICC: Immunocytochemistry; IHC: Immunohistochemistry; NA: Not available; RDOR: Relative diagnostic odds ratio; SE: Standard error; TTF-1: Thyroid transcription factor 1;
