# Supplementary material for: Diagnostic value of thyroid transcription factor-1 for pleural or other serous metastases of pulmonary adenocarcinoma: a meta-analysis
Source: Sci Rep. 2016 Jan 25;6:19785. doi: 10.1038/srep19785 (PMC4726262; doi:10.1038/srep19785)
Supplement: Supplemental Table 1 [file srep19785-s1.pdf]

Diagnostic value of thyroid transcription factor-1 for pleural or other serous metastases of pulmonary adenocarcinoma: a meta-analysis

Yongchun Shen[1#], Caishuang Pang[1#], Konglong Shen[2], Yanqiu Wu[1], Diandian Li[1], Chun Wan[1], Zenglin Liao[1], Ting Yang[1], Lei Chen[1], Fuqiang Wen[1*]

1. Department of Respiratory and Critical Care Medicine, West China Hospital of Sichuan University and Division of Pulmonary Diseases, State Key Laboratory of Biotherapy of China, Chengdu 610041, China.

2. Radiation Physics Center, Cancer Center and State Key Laboratory of Biotherapy, West China Hospital of Sichuan University, Chengdu 610041, China.

# Equal contributors

*Correspondence to:

Fuqiang Wen M.D.

E-mail: wenfuqiang.scu@gmail.com

Tel: +86-28-85422350 Fax: +86-28-85582944

Author (Ref	Year	Samples	Number	Gastrointestinal															
				Breast	Gastric	Colon	Rectum	Mixed	Ovary	Pancreas	Endometrium	Esophagus	Bile duct	Prostate	Gallbladder	Liver	Thyroid	Kidney	Other
Hecht JL (15)	2001	PE,PTE,PAE	49	18	3				16	2	4	3		2	1				
Jiang KY (16)	2001	PE,PTE	40	8	9	8			15										
Afify AM (17	2002	SF	36	12				12	12										
Gomez-Fernai	2002	PE,PTE	74	24	3	5			25	6				1	1				9
Ng WK (19)	2002	PE,PTE,PAE	19		8	5			4		1		1						
Su X (20)	2004	PE,PTE,PAE	24	2				3	12	1	1	3		1	1				
Jan IS (21)	2006	PE,PAE	25	5	11	2*			6							1			
Dejmek A (22	2007	PE	20	8	2	1				1	1								7
Zhou C (23)	2007	PE	11	3	4	1			2		1								
Zhu W (24)	2007	SF	33	13					20										
Jiang B (25)	2008	PE	5	2	1				2										
Wang J (26)	2008	PE	18	12				5									1		
Kim JH (27)	2010	PE,PTE	45	3	28	2			6	4			2						
Khoor A (28)	2011	PE	26	13	2	1			5	1		1		2				1	
Kim JH (29)	2011	PE	31	9	9		1		5	1			5		1				
Liu L (30)	2012	PE	15	13					1			1							
Liu Y (31)	2012	PE,PTE,PAE	20	9	2	4			4	1									
Luo Q (32)	2012	PE,PTE	16					6	10										
Yan J (33)	2014	PE	25	14	9														2
Yin J (34)	2014	PAE	13	10						3									
		Summary	545	178	91	29	1	26	145	20	8	8	8	6	4	1	1	1	18

* means colorectal cancer
